# Utilizing river and wastewater as a SARS-CoV-2 surveillance tool to predict trends and identify variants of concern in settings with limited formal sewage systems

**DOI:** 10.21203/rs.3.rs-2801767/v1

**Published:** 2023-04-14

**Authors:** Kayla Barnes, Joshua Levy, Kristian Andersen, Jillian Gauld, Jonathan Rigby, Oscar Kanjerwa, Christopher Uzzell, Chisomo Chilupsya, Catherine Anscombe, Christopher Tomkins-Tinch, Omar Mbeti, Edward Cairns, Herbert Thole, Shannon McSweeney, Marah Chibwana, Philip Ashton, Khuzwayo Jere, John Meschke, Peter Diggle, Jennifer Cornick, kondwani Jambo, Gift Kawalazira, Steve Paterson, Tonney Nyirenda, Nicholas Feasey, Benjamin Chilima

**Affiliations:** Harvard TH Chan School of Public Health; Scripps Research Institute; Department of Immunology and Microbiology The Scripps Research Institute La Jolla CA USA; Institute for Disease Modeling, Bill & Melinda Gates Foundation; Department of Clinical Sciences, Liverpool School of Tropical Medicine; Malawi-Liverpool-Wellcome Clinical Research Programme, Kamuzu University of Health Sciences, Blantyre, Malawi; Malawi-Liverpool-Wellcome Clinical Research Programme, Kamuzu University of Health Sciences, Blantyre, Malawi; Malawi-Liverpool-Wellcome Trust Clinical Research Programme; Broad Institute of MIT and Harvard; Blantyre District Health Office; Department of Evolution, Ecology and Behaviour, Institute of Infection, Veterinary and Ecological Sciences, University of Liverpool; Malawi-Liverpool-Wellcome Clinical Research Programme, Kamuzu University of Health Sciences; Department of Clinical Sciences, Liverpool School of Tropical Medicine; Malawi-Liverpool-Wellcome Clinical Research Programme, Kamuzu University of Health Sciences; Malawi Liverpool Wellcome; University of Liverpool; University of Washington; Lancaster University; Department of Evolution, Ecology and Behaviour, Institute of Infection, Veterinary and Ecological Sciences, University of Liverpool; Liverpool School of Tropical Medicine; Blantyre District Health Office; University of Liverpool; Department of Pathology, Kamuzu University of Health Sciences; Liverpool School of Tropical Medicine; Public Health Institute of Malawi

## Abstract

The COVID-19 pandemic continues to impact health systems globally and robust surveillance is critical for pandemic control, however not all countries can sustain community surveillance programs. Wastewater surveillance has proven valuable in high-income settings, but little is known about how river and informal sewage in low-income countries can be used for environmental surveillance of SARS-CoV-2.

In Malawi, a country with limited community-based COVID-19 testing capacity, we explored the utility of rivers and wastewater for SARS-CoV-2 surveillance. From May 2020 – January 2022, we collected water from up to 112 river or informal sewage sites/month, detecting SARS-CoV-2 in 8.3% of samples. Peak SARS-CoV-2 detection in water samples predated peaks in clinical cases. Sequencing of water samples identified the Beta, Delta, and Omicron variants, with Delta and Omicron detected well in advance of detection in patients. Our work highlights wastewater can be used for detecting emerging waves, identifying variants of concern and function as an early warning system in settings with no formal sewage systems.

## Introduction

As the COVID-19 global pandemic continues, it has become apparent that globally representative tracking of disease trends and rapid identification of novel variants is essential to pandemic control^1^. Whilst high-income countries have high rates of vaccine coverage, including booster campaigns, LICs lag in comparison, creating environments where severe acute respiratory syndrome coronavirus 2 (SARS-CoV-2) can continue to circulate and mutate, especially in settings with high prevalence of immunosuppressive illness^2,3^. In Malawi, there is limited capacity within the health care system to carry out large-scale surveillance of COVID-19 in the community, especially between waves, which limits the ability of public health services to detect waves early as hospitalization lags community amplification. An early warning system to detect increasing cases and identify imported or novel variants of concern (VOCs) is therefore urgently required.

Genomic surveillance of SARS-CoV-2 is currently limited to two Malawian cities (Blantyre and Lilongwe) and is limited due to low sequencing resources and capacity. In Malawi, sero-surveys have shown high levels of exposure consistent with high income countries, with 27.55% of healthcare workers testing positive for anti-SARS-CoV-2 antibodies as early as 2020^4^. Further, in 2021 over 80% of local blood bank samples were positive for antibodies^5^. There have also been high levels of asymptomatic infection (45.7%)^6^. These studies show vastly higher circulation of SARS-CoV-2 than reported through surveillance of individuals presenting with clinical features of COVID-19 to secondary or primary care facilities. Although there is high exposure in Malawi providing some population-level immunity by the end of 2022 only around 13% of the population has been vaccinated^7^ against COVID-19, leading to a population at greater risk for circulating VOCs. The disparity between genomic surveillance and clinical caseloads has created a need for novel surveillance strategies that can survey a larger proportion of the population at low enough cost to be sustainable, yet which offer the resolution to predict trends in clinical disease and identify emerging VOCs.

The utility of wastewater surveillance to identify VOCs and track COVID-19 has been well established^8–10^. This method takes advantage of high SARS-CoV-2 concentrations in feces, typically in the range of 10^3 to 10^7 RNA copies per gram, in more than 50% of symptomatic patients^11^, further fecal viral shedding can continue even after symptoms subside^12^. SARS-CoV-2 RNA shed into wastewaters can therefore be detected, quantified, and sequenced from wastewater to monitor transmission, estimate prevalence, and track variants^13–16 17,18^. Finally, recent advances in computational analysis of wastewater samples allows for both clinically observed lineage and cryptic lineage detection, i.e., virus lineages that are not being detected in the human population but are still circulating^19^.

Globally, only 52% of sewage and wastewater is treated and this drops to only 4.2% in low-income countries (LICs)^20^. In Blantyre, the 2nd largest city in Malawi there are no active sewage treatment plants. Instead, the population largely uses earthen (67%) or concrete (20%) latrines which often lead to fecal contamination of groundwater and river systems. Thus, sanitation infrastructure deficit is common to many LICs and it is unclear whether and how surveillance of the contaminated environment (henceforth environmental surveillance or ES) can be used as a public health tool to track SARS-CoV-2 and inform decision and policy makers^13,21,22^. Our recent work highlighted how ES can be a cost-effective tool in LICs^23^ and here we describe the full utility of river and wastewater surveillance.

## Results

### SARS-CoV-2 detection in river and sewage

**Figure 1:**
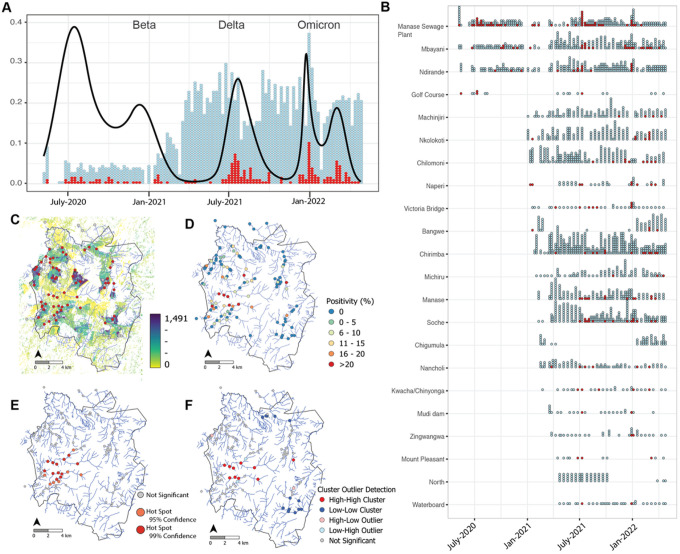
Temporal ES sampling in Blantyre Malawi. A) sampling overtime where each individual dot is one sample tested either negative (blue) or positive (red) for SARS-CoV-2. The y-axis and black line are the Spline curve modeling peaks and valleys in detection based on the frequenting of positivity. B) The region-specific dot plot shows 22 areas of Blantyre sampled during phase 1 (2020) and/or phase 2 (2021–2022) by negative (blue) or positive (red) for SARS-CoV-2. This also shows there was over sampling and under sampling of some regions of the city and regions with higher SARS-CoV-2 detection. C) Red dots denote sites with at least one positive sample overlaid on the population density of Blantyre based on HRSL data. D) each sampling location is color coded by the overall percent positivity based on the full collection. E) Hotspot analysis using Getis-Ord G_i_* and F) spatial cluster-outlier detection analysis using Anselin Local Moran’s *I*. Utilizing this analysis sites can be reduced for future collections to less than 20 sites.

Utilizing our previously established ES program for *Salmonella enterica* serovar Typhi^24,25^ we based our ES for SARS-CoV-2 on 30ml “grab” water samples. Water from rivers, informal sewage systems and a defunct wastewater treatment plant was collected in two phases; from May 2020 – January 2021 we developed our sample framework, field sampling method and laboratory methods and from January 2021 – May 2022 we greatly expanded the scale of our program^26^ (Fig. 1A). Phase 1 included collections in 7 sites spanning 3 urban areas of Blantyre, Malawi, and Phase 2 scaled the collection to 112 sites covering 22 areas representing river catchment areas of diverse population size in Urban Blantyre (Fig. 1B, Table S1).

In Phase 1, we tested water effluent for SARS-CoV-2 by PCR after filtering through a 0.45-micron filter for SARS-CoV-2. After comparing filtered samples to unfiltered grab samples, we found no difference in SARS-CoV-2 detection (Fig S1A). We also compared two widely used concentration methods, polyethylene glycol (PEG)^27^ and skimmed milk flocculation (SMF)^27,28^ concentration and found no significant difference in diagnostic outcome (Fig S1B). We chose grab samples due to cost and straightforwardness in the field and PEG due to more predictable availability of consumables and laboratory infrastructure.

We first detected SARS-CoV-2 on May 11th, 2020, and from May 2020 to May 2022, we found 8.3% (220/2625) of our samples were positive for SARS-CoV-2 by PCR, with Ct range of 30–39.45 (Fig S1C). Sampling rates and positive detections varied over time (Fig. 1A) and by site (Fig. 1B–D). Sewage samples had higher SARS-CoV-2 positivity rates (21% [55/258]) than river water samples (7.2% [170/2367]), however river water samples still yielded a signal which varied in line with the occurrence of new waves of COVID-19 in Blantyre. One or more sample(s) had detectable SARS-CoV-2 at 42/112 sites (Fig. 1B–D). Using Anselin Local Moran’s *I* and Getis-Ord G_i_* analysis we identified statistically significant high-high clusters and hotspots of positivity, respectively. Specifically, increased rates of detection were geographically centered toward the southwest of Blantyre along the Mudi and Naperi rivers which drain through the most densely populated areas of the city (Fig. 1E–F). Moreover, local Moran’s *I* also identified statistically significant low-low clusters of positivity in the southeast and north of Blantyre indicating areas of significantly low rates of detection. This has allowed us to decrease sample collection sites to 21 highly informative locations that demonstrate clear waves of positivity in ES data, and provide representative surveillance for around 70%, and of the population.

### Validation of ES as a predictor of clinical prevalence

Next, we aimed to validate ES as a predictor of clinical cases in the surveillance system. The clinical dataset we utilized was from an active recruitment study, where from November 2020 to July 2021, a maximum of 20 individuals/day who met the WHO syndromic definition of COVID-19 were tested. From August 2021, a maximum of 21 patients per site per weekday were recruited, at a 2:1 ratio of those who were suspected cases and those who were not, to capture asymptomatic transmission^29^. The active community surveillance program tracked both symptomatic and asymptomatic cases as well as number of diagnostic tests conducted. Using a quasi-binomial generalized linear model, we found that the patterns of SARS-CoV-2 detection in ES samples significantly predict the rate of positivity in the community surveillance system for all waves (p < 0.001) (Table 1). The estimated intercepts in the model can inform the sensitivity of the ES system, and we find an ES detection rate of zero corresponds with approximately 5–6% prevalence in the community in the Delta and Omicron waves, indicating lack of sensitivity of the ES system below this prevalence rate. As COVID-19 increased in the community, with each wave driven by a new VOC, so did detection of SARS-CoV-2 in wastewater.

Further, we allowed for wave-specific lagged effects. For the Beta wave, we found that the best fit of the model resulted in the ES predating peak prevalence by almost two months (Fig S2). Other COVID-19 waves were more closely aligned with ES data (Fig S2). Further, we detected differences in wave-specific sensitivities of the ES system (Table 1, Fig S3). A 20% detection rate in ES during the beta-wave corresponded to a 50% prevalence rate in the community. The same rate of detection in ES would correspond to a 30% prevalence rate if it occurred in the delta wave, indicating a possible decline in healthcare seeking or reporting in the second wave.

Finally, we tested the robustness of these estimates to uncertainty in the ES data. We generated 1,000 realizations from the multivariate normal distribution parameterized by the ES regression model covariates (Fig S4). The realizations were significant (p < 0.05) for 100% and 99.9% of iterations for Delta and Omicron, respectively, and 92% for the Beta wave (Fig S5), reflecting uncertainty in the first wave’s predictive power based on fewer samples during this early time period.

### Comparison of the peaks of SARS-CoV-2 from ES in relation to clinical datasets

**Figure 2:**
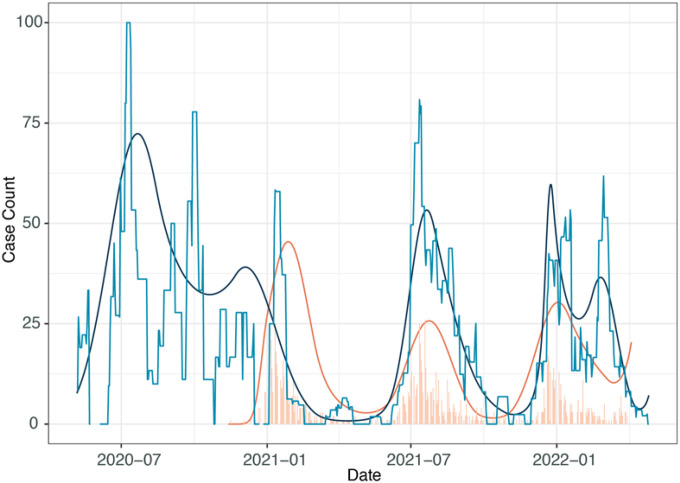
Estimates of lag time between ES and active case surveillance. Light blue represents the rolling average of ES positivity over time compared to active case surveillance numbers (bar graph in light orange) where the y-axis is total on number of positive cases per day. Spline comparison of both ES (dark blue) and active case surveillance prevalence (dark orange) show how closely linked peaks in both detection methods are over multiple waves.

We next wanted to compare peaks of SARS-CoV-2 detections across datasets. We utilized a secondary dataset reflecting clinical patterns of COVID-19, data reported to the Blantyre District Health Office (DHO). This dataset contained daily counts of reported city-wide cases and was available from the beginning of ES sampling until January 2022, and represented case detection from symptomatic patients seeking treatment and limited contact tracing. A large proportion of individuals presented to secondary community health centers which is representative of data more typically available in low-income settings. There were key limitations to this dataset. The DHO dataset did not record the total number of tests administered and there was limited testing of asymptomatic individuals mainly at the beginning of the pandemic. In addition, there were periods of low testing due to PCR resources and there was a national switch from PCR based diagnostics to rapid diagnostic tests, but this was not captured in the dataset available. Examining this data, the Delta wave appears significantly smaller than the other waves, with the Omicron wave the largest (Fig S6), and these data were thus distinct from the ES and community-based active surveillance data (Fig. 2).

We compared the timing of the peak of COVID-19 rates across all three datasets. The reported DHO positivity data lagged behind ES data by 2, 31, and 7 days for Wave 1, Beta wave and Omicron wave, respectively, however for the Delta wave the ES data lagged behind the DHO data by 13 days (Table 2). Despite the differences between active surveillance, passive surveillance and ES, the peaks of the waves were very similar, further validating ES as a useful indicator for COVID-19 outbreaks.

### SARS-COV-2 sequencing and identification of variants of concern.

To better understand if ES can be used to identify VOCs circulating in river and informal sewage, we carried out amplicon sequencing (see Methods) using the Nanopore Minion for 90 samples with the lowest diagnostic cycle threshold (Ct) between 30.2–38.8, of which 85 produced sequences. Although this is now commonly done HIC with refined sewage systems it was unclear if we could identify VOCs from very mixed samples with low viral loads. Of our sequenced samples, only 24/86 samples had > 50% coverage and 52/86 samples had > 20% coverage at 20x depth. To confirm our sequencing results and compare sequencing methodologies we carried out further matched sequencing of 68 samples using the EasySeq method (see Methods) and an Illumina MiSeq or NovaSeq (Fig S7). Of these samples, 27/68 had > 50% coverage and 42/68 samples had > 20% coverage at 5x depth showing that low viral load greatly affected both sequencing methods.

**Figure 3:**
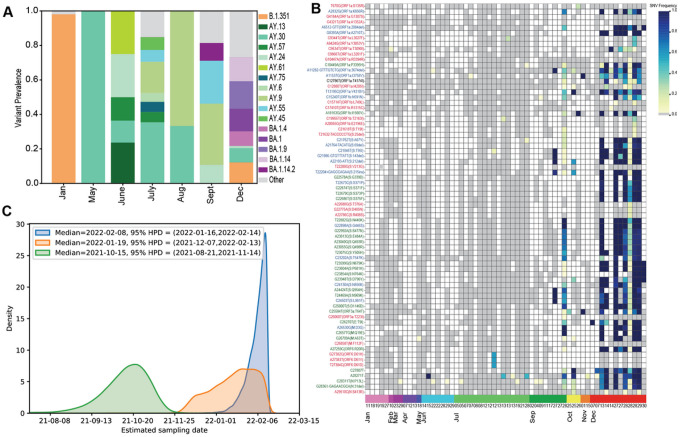
SARS-CoV-2 variant in wastewater identified key VOCs before observed in the patient population. A) Summary of VOC detected by month using Freyja, B) Omicron SNPs show putative early detection of the VOC. The heatmap shows all Omicron SNPs on the y-axis (blue=BA.1-specific, red=BA.2-specific, green=BA.1/2 shared mutation) and individual ES samples overtime on the x-axis where months are by color. In September (samples have some key Omicron SNPs but lack the full repertoire of SNPs which become dominant by December. C) Time-calibrated Bayesian phylogenetic analysis of the early Omicron samples under three considerations: genome assembly with only physically linked mutations (green), genome assembly with the inclusion of the frequency-linked BA.14 mutation (orange) and only high frequency mutations (blue). This analysis showed higher confidence in the mid-January genome.

We were able to sequence SARS-CoV-2 from both informal sewage and river water, important for ES in communities with limited to no refined sewage treatment centers. We utilized the Freyja analysis tool^19^ to identify circulating VOCs (Fig. 3A). We first identified VOCs on the following dates: Beta (Jan 19th, 2021), Delta (May 18th, 2021) and Omicron (28th, Sept 2021) using both Minion and Illumina data. Detection of Beta was consistent with clinical data; however, Delta was detected in wastewater a week before the first clinical detection in Blantyre (May 26th, 2021). Our initial analysis identified Omicron over two months before we observed a clinical case of Omicron in Malawi (Dec 6th, 2021), and 6 weeks before the first identification of this new variant globally (Nov 9th, 2021)^30,31^. Freyja analysis uncovered some examples of cryptic transmission where Beta continues to pop up throughout the year and Omicron does not fully replace Delta until December (Fig. 3A) which was not identified in our patient data.

At face value the two genomes from September appeared to be Omicron by Freyja and one was identified as Omicron using the ARTIC to Pangolin computational pipeline^31^. Upon deeper analysis of the two putative Omicron genomes from wastewater samples collected in September 2021, we found that one majority consensus genome had multiple SNPs found in a clinical case (a BA.1.14 lineage virus) from Malawi collected in January 2022 and sequenced a few weeks before our wastewater sample was sequenced (Fig S9A). Although concentration, extraction and library preparation were carried out in an ES only lab the amplicon sequencing was carried out in a shared lab space. We re-sequenced the wastewater sample from the original water sample and the re-sequence did not match the clinical sample and did not have the full Omicron repertoire of SNPs (Fig S9B), confirming our suspicion of contamination. The re-sequenced sample contained key Omicron SNPs including specific to BA.2 and BA.4, which is not in line with the putative early Omicron signatures (Fig S9B).

For the second putative Omicron positive sample from September 2021, we further investigated how to analyze a sample with two VOCs present. For this sample Omicron was estimated to be only about 10% of total virus present, we attempted to produce a unique genome by leveraging physically linked mutations specific to Omicron, SNP-frequency linkage (i.e., similar mutation frequency) and by filtering out reads corresponding to circulating Delta lineages, the major VOC in our sample. We were able to generate an Omicron consensus genome but at low sequencing coverage and many Omicron SNPs were either missing or at frequencies under 50% (Fig. 3B). We further scrutinized this genome to better define if the phylogenetic timestamp of this genome was indicative of an Omicron precursor. Since genomes from wastewater are a mixture of multiple viruses calling a consensus genome is much more complicated and minor decisions (e.g., physically linked vs unlinked SNPs, frequency of each SNP, classifying SNPs that are linked to multiple VOC, etc.) can greatly change the time-aware phylogeny (Fig. 3C). Using a Bayesian phylodynamic approach, we tested the effect of three different consensus calling approaches on the estimated sampling date of the virus (i.e., while blinding our model to the “known” sampling date of the virus). If we included all physically linked and SNP frequency-linked mutations, we estimated sampling date in early 2022 (median Feb 8, 95% HPD = (Jan 16-Feb14)), in line with the global spread of Omicron and not indicative of early emergence in Malawi.

However, since Omicron was identified as a minority component in the mixture, our initial consensus approach may incorrectly include additional mutations associated with Delta lineages at a comparable frequency. Hence, we considered the inclusion of two additional cases, corresponding to using only physically linked mutations and only physically linked mutations plus the frequency-linked C12786T (Orf1a:T4174I) mutation (a lineage-defining mutation for BA.1.14, which later circulated in Malawi). When considering only physically linked mutations we found a median estimated sampling date of October 15th, 2021 (95% HPD= (Aug 21, Nov 14) consistent with early Omicron circulation in Malawi prior to November 2021. Although we cannot rule out that our retrospective detections of Omicron in September 2021 constitute *bona fide* early evidence of this VOC, given our combined findings, we believe it is more likely than not that our September 2021 detection could be due to laboratory contamination during sequencing thus highlighting the importance of real-time sequencing.

To understand how the ES sample genomes relate to patient data from Malawi as well as SARS-CoV-2 more globally we constructed maximum likelihood phylogenetic trees for Delta and Omicron. We utilized consensus genomes derived from the Freyja analysis that had greater than 50% estimated Omicron abundance. We built trees utilizing patient derived genomes from a time period similar to our samples with a bias toward genomes from Africa. For Delta the ES samples largely cluster with genomes from patients’ samples from Malawi and samples from South Africa (Fig S9).

Next, we sought to understand cryptic transmission of VOCs even with low samples and low coverage. Among samples with ≥ 20% genome coverage 15/51 contained more than one VOC and for samples with ≥ 50% genome coverage 5/28 samples contained more than one VOC. This timeline of cryptic detection and then the emergence of dominant VOCs is consistent with sequencing data from patients in Malawi where new VOCs become dominant in the patient population. Although we have low numbers and low genome coverage, we do see examples of cryptic transmission of known VOCs. We detect Beta intermittently until the end of 2021 and we continue to detect Delta even after Omicron moved through the population (Fig. 3A). Our current patient genomic surveillance failed to detect the continued circulation of these VOCs^30^.

## Discussion

We demonstrate that environmental surveillance of SARS-CoV-2 has the potential to be an important public health tool in a low-income setting where there is often no formalized sewage system. We detected SARS-CoV-2 as soon as we started sampling in May 2020. We describe city-wide detection of SARS-CoV-2 in river systems for the first time, highlighting any source of water with fecal contamination can be used for SARS-CoV-2 detection. This demonstrates communities without adequate sanitation systems can sample rivers and semi-formal sewage systems to generate actionable information about SARS-CoV-2 trends. We initially oversampled Blantyre, using multiple locations to capture a high percentage of the population and determine where virus was accumulating. Using hotspot and cluster analysis we were able to decrease our collection to around 20 maximally informative sites that cover around 70% of the population, greatly reducing the overall cost of this surveillance. We propose this inexpensive approach can run in parallel with sentinel community surveillance to provide an early warning system for public health officers to scale surveillance and public health interventions in response to environmental detection.

Rates of sample positivity correlated with clinical prevalence of COVID-19 in Blantyre, Malawi. Peaks of positivity in our ES samples preceded peaks in cases in the population when compared to both active and passive surveillance approaches except for during the Delta wave which showed similar trends between all datasets. This is consistent with trends observed in wealthier countries based on wastewater surveillance of refined sewage systems where early warning signals hover around 2 weeks^9,32^. Our modeling shows that increases above 5% positivity in the ES data continue to trend upward and are predictive or at the very least mirror increases in the population. Utilizing this early warning signal community health centers and hospitals can scale supplies and staff to counteract the total burden on the healthcare system. In addition, public service announcements can be used to try and minimize close contacts and therefore decreases surges in the population and protect vulnerable people.

We demonstrate that genomic analysis from rivers and informal sewage is possible. Prior to the COVID-19 pandemic there had been no sequencing in Malawi, and this work helped establish genomic surveillance within the country. Whilst we were able to detect diverse VOCs from wastewater, our experience highlights the complexities of consensus calling for mixed samples, especially as new VOCs arise while older viral lineages are still circulating. This work established Beta, Delta, and Omicron VOCs circulating in the Southern region of Malawi, but our work is also a cautionary tale on the level of scrutiny needed to identify emerging VOCs. Our work identified Beta and Delta using almost real-time sequencing carried out prior to patient sample sequencing. Our Omicron detection presents a much more complicated genomic analysis. We first identified Omicron circulation prior to its detection in South Africa utilizing the two standard pipelines for calling a consensus genome (Freyja and PANGOLIN) but in-depth phylogenetic analyses established clear contamination of one sample and our second Omicron genomes was < 10% of the total sample and contained SNPs indicative of more derived Omicron. Despite significant efforts to separate processing of environment and clinical samples, pathogen sequencing of these samples was not performed in real-time, making it diffi cult to distinguish between actual early VOC detection and contamination. Our work does provide key analysis steps to disentangle mixed samples and create consensus genomes from VOCs at low percentage. As many VOCs will originate in countries will low genomic surveillance, early detection in wastewater could be a powerful tool but sequencing needs to be carried out in real-time, both to enable timely public health interventions and to limit confounders for analyses, an issue that has plagued multiple SARS-CoV-2 genomic surveillance studies even from clinical samples.

ES has clear potential to act as an inexpensive^23^ early warning system in the surveillance of SARS-CoV-2 in low-income communities with limited community surveillance. Further, it has the potential for multiplexing to identify other environmentally dependent pathogens as we’ve shown through the detection of *S. Typhi* from matched water collection samples. This can be expanded to other pathogens of public health importance such as Polio and Rotavirus as well as other enteric viruses and bacteria at minimal extra cost. ES is a rapid and cost-effective tool to predict peaks in transmission and follow the introduction of VOCs. Whilst future work must explore the impact of the water conditions (pH, organic composition, flow rate), sampling strategies, concentration, sequencing methods and timing and finally robust computational pipelines, ES is already an effective solution to tracking outbreaks and re-emergence of long-standing viral threats.

## Methods

### Ethics

Wastewater samples, although not human subjects, were collected under the ethical waiver P.07/20/3089 from the College of Medicine Research Support Centre (CoMREC). No identifiable information was used for the estimations of detection frequency between ES and the population. Nevertheless, active surveillance data was collected under CoMREC P.08/20/3099 and the Liverpool School of Tropical Medicine Research Ethics Committee (LSTMREC 21–058). Passive surveillance was collated by the District Health Office and no identifiable information was used for this analysis.

### Sampling strategy

For 2020 we utilized collections sites that we already had an ongoing *S Typhi* surveillance program. Once we established, we could detect SARS-CoV-2 in river and informal sewage we scaled our collection to 112 sites spanning 22 areas of Blantyre, Malawi (Fig. 1B). Sample sites were chosen using a systematic GIS-based framework utilizing remote sensed environmental data coupled with a novel spatial analysis^25^. In Brief: we identify all potential candidate ES sites based on the location of hydrological (river) confluence points based on the assumption that a natural converging river network serve a large percentage of the population. Hydrological catchments were delineated for all candidate ES sites and we used the High Resolution Settlement Layer (HRSL) (https://ciesin.columbia.edu/data/hrsl - last access: 27 March 2021) population dataset and catchment geomorphology characteristics to guide and inform the best site locations. Next, we systematically removed ES sites considered unsuitable by cross referencing with spatially explicit land use datasets to identify with low population density.

Samples from each location were collected weekly from May 14th - Dec 18th, 2020 (phase 1) with gaps in the collection largely due to safeguarding of the field team as the COVID-19 pandemic unfolded in Malawi. During Phase 2 we started collecting Jan 3rd, 2021, with full scale up by mid-February, 2021 of 112 sites collected about every 2 weeks or as needed due to changes in river dynamics and safety of the field team (40–50 sites/week) equally around 90 samples/week (Fig. 1B, Table S1). If a site was deemed unsafe to collect either due to heavy rains, access, or personal safety they were either eliminated or missed until the site became safe again.

### Concentration of samples

We adopted polyethylene glycol (PEG) concentration methods based on work by Philo et al.^33^ Briefly, we added 3gram of PEG and 0.68gram of NaCl to a 30mL grab sample in a 50mL Falcon tube. We manually shake by hand for 30 seconds or until the PEG and NaCl is dissolved. Up to 20 samples are then spun at 1200g, 4°C (800–2000g is ok though) for 2 hours. We did test decreasing the spin time to one hour and see little difference in SARS-CoV-2 detection. After centrifugation we discard supernatant and then add 200–300uL sterile PBS (PH 7.4) to the pellet and vortex for 2 minutes for a final volume of 500ul. Samples were then transferred to a (locking) 1.5 Eppendorf tubes or cryovials and stored at −80°C prior to RNA extractions.

### SARS-CoV-2 detection

PEG samples were extracted using either the Qiagen RNA viral extraction kit or the Qiasymphony-DSP mini kit 200 (Qiagen, UK) with offboard lysis. RNA was tested for SARS-CoV-2 using the CDC N1 assay (IDT) and qScript 1-step master mix (10ul master mix, 1.5 primer/probe (FAM), 3.5ul water, and 5ul RNA) with a positive and negative control. We also ran a standard curve using a serial dilution of a SARS-CoV-2 genomic fragment from 1,000,000 to 10 copies/ul.

### Statistical methods for SARS-CoV-2 from ES

To model temporal trends in ES positivity rate, positive/ negative ES detection rates were incorporated into a model using logistic GLM, including a b-spline to capture temporal trends in detection rates. Knots were iteratively selected to minimize the binned weekly Pearson residuals, which were visually assessed by generating plots of weekly residuals over time.

We model the case data as a quasi-binomial GLM to correct for overdispersion in the binomial GLM and use the modeled ES detection rate as a predictor. We know that there may be wave-specific differences in ES as it relates to clinical prevalence, due to changes in the sensitivity of the ES system, the relative amplitudes between waves (driven by healthcare seeking/ symptom severity differences) and delay between shedding and healthcare seeking (timing of symptoms), therefore we allow for wave-specific intercepts and lags.

In order to estimate the wave-specific lags for the combined model, we first model each wave individually using the following equation:

$$\begin{array}{l} Y_{t}\sim \text{Quasi Binomial}\left(u_{t}\right)\\ \text{log}\left(\frac{u_{t}}{1-u_{t}}\right)=\alpha +\beta *ES_{t-lag} \end{array}$$

We investigated lags from − 21 (3-week lead time) to 56 (8 weeks). Finally, we include the lagged ES time series for each wave in a full model:

$$\begin{array}{l} Y_{t}\sim \text{Quasi Binomial}\left(u_{t}\right)\\ \text{log}\left(\frac{u_{t}}{1-u_{t}}\right)=\overset{3}{\underset{w=1}{\sum }}\left(\alpha_{w}+\beta_{w}*ES_{t-\text{ lagw}}\right)*Z_{w,t} \end{array}$$

*Z*_*w*,*t*_ is an indicator variable that is equal to 1 when t is contained in wave w, and 0 otherwise. We additionally tested the robustness of these estimates to uncertainty in the ES time series. We generated 1,000 realizations from the multivariate normal distribution parameterized by the ES regression model covariates. Next, we tested the significance of these realizations as predictors in the full model and summarized the significance of the covariate across this uncertainty.

### Comparing peaks between datasets

An additional dataset was obtained by the Blantyre DHO and summarized daily counts of reported city-wide cases from the beginning of ES sampling until September 2021. Along with the other two datasets informing covid incidence over time, we summarized the timing of each peak in the dataset, by fitting a b- spline to each time series and extracting the timing of the maximum values. To account for uncertainty in the time series, we generated 1,000 realizations from the multivariate normal distributions parameterized by these models.

### Sequencing and analysis of SARS-COV-2

For positive samples ct < 39 cDNA was generated using a 2-step process using Superscript IV (ThermoFischer) or a 1-step process and using LunaScript (NEB). For minion sequencing carried out in Malawi we utilized a modified ARTIC sequencing protocol V2. From May 2020 - December 2021 ARTIC V3 primers were used and in January 2022 we switched to V4 primers when they became available for order. Matched samples were also sequenced using an adopted EasySeq method (https://www.protocols.io/view/wastewater-sequencing-using-the-easyseq-rc-pcr-sar-81wgb7bx3vpk/v2 and https://www.nimagen.com/gfx/Covid19/protcol-NimaGen-covid-wgs_v201.pdf). When possible, we generated new cDNA using the EasySeq method which has a concentration step before cDNA generation. We used a 1.8x SPRI beads (Beckman Coulter) We found this yielded higher cDNA per ul. If we could not regenerate the cDNA using the EasySeq protocol, we utilized cDNA generated for the original minion sequencing. After cDNA generation the Easyseq method was carried out using either a V3 or V4 amplicon primer set.

For minion sequencing analysis we adapted the CLIMB/ARTIC analysis pipeline to be carried out locally^31^. Briefly, FAST5 data was processed using Guppy v5.0.7 including guppy_basecaller. Bam files were generated from FASTQs by assigning barcodes using guppy_barcoder, eliminating sequences lacking barcodes at both ends and using medaka ARTIC field bioinformatics pipeline. Available at: https://github.com/artic-network/fieldbioinformatics). Finally, consensus genome (FASTA) files were generated using the original Wuhan genome based on the ARTIC pipeline^31^.

For the VOC per sample analysis, we utilized the Freyja methods^19^ which incorporates viral genetic diversity and infers relative abundance of lineage-defining mutations to deconvolute mixed samples. We used .bam files from both the minion and Illumina sequencing and the Freyja workflow and packages found at https://github.com/andersen-lab/Freyja.

We performed consensus calling only on samples including > 50% Omicron (as estimated by Freyja) and called SNVs with 50% or greater frequency. We then aligned genomes specific to the Delta and Omicron VOCs separately and analyzed these with genomes from a similar time range from patient samples derived from Malawi, elsewhere in Africa, as well as samples from around the world. We aligned to reference using minimap2^34^ with gofasta^35^ and used *masking* to remove homoplasic sites (https://virological.org/t/masking-strategies-for-sars-cov-2-alignments/480). Maximum likelihood tree inference was generated using IQ-TREE2^36^ and tree rooting, and visualization were done using the toytree package. Heatmap was generated utilizing all known SNPs associated with the Omicron BA.1 and BA.2 lineages.

Bayesian date sampling analyses for putative early-Omicron samples was done using BEAST 1.10.4^37^. We used an HKY substitution model with gamma-distributed rate variation, a strict molecular clock, and an exponential growth coalescent tree prior. Analyses were performed on a background set of 380 early Omicron BA.1 sequences of high quality. Date sampling was performed using an uninformative uniform prior for the sample collection date. For date sampling of each candidate consensus sequence, we performed 200 million Markov Chain Monte-Carlo steps, with at least the first 20% of steps discarded as burn-in.

## Figures and Tables

**Table 1:** Estimated model parameters for the predictive model of Covid cases showing wave-specific coefficients for the ES rate as a predictor (α), as well as wave-specific intercepts (β) denoted for each wave driven by a VOC.

	Estimate	Std. Error	Pr(>|t|)
*α*_*Beta*_	−4.65926	0.493613	6.84E-19
*α*_*Delta*_	−2.90824	0.16818	3.18E-48
*α*_*Omicron*_	−2.77117	0.203336	6.73E-34
*β*_*Beta*_	23.32583	2.950117	3.95E-14
*β*_*Delta*_	10.2418	0.893807	7.72E-26
*β*_*Omicron*_	11.40473	1.196987	3.50E-19

**Table 2:** Estimated date of peak detection. Timing of the peak of each wave between datasets shows both active and passive detection lags behind ES detection except during the Delta wave.

Wave (VOC)	Environmental Surveillance	Community Active Surveillance	Community passive Surveillance (DHO)
Wave 1	7-13-2020 (5-28-2020, 8-01-2020)	NA	7-15-2020 (7-14-2020, 7-17-2020)
Wave 2 (Beta)	12-05-2020 (10-29-2020, 12-23-2020)	1-27-2021 (1-22-2021, 1-31-2021)	1-05-2021 1-04-2021, 1-06-2021)
Wave 3 (Delta)	7-22-2021 (7-14-2021, 8-04-2021)	7-22-2021 (7-19-2021, 7-25-2021)	7-09-2021 (7-08-2021, 7-12-2021)
Wave 4 (Omicron)	12-28-2021 (12-22-2021, 3-07, 2022)	12-30-2022 (12-26-2021, 1-05-2022)	1-04-2022 (1-03-2022, 1-05-2022)

## Data Availability

All raw sequence data are available at the NCBI Sequence Read Archive under BioProject ID PRJNA887942: ES SARS-CoV-2 Malawi. GPS coordinates of collection sites are available upon request.
